# Milk-exosome based pH/light sensitive drug system to enhance anticancer activity against oral squamous cell carcinoma

**DOI:** 10.1039/d0ra05630h

**Published:** 2020-07-29

**Authors:** Qian Zhang, Qi Xiao, Honglin Yin, Chengwan Xia, Yumei Pu, Zhifeng He, Qingang Hu, Jianquan Wang, Yuxin Wang

**Affiliations:** Department of Oral and Maxillofacial Surgery, Nanjing Stomatological Hospital, Medical School of Nanjing University Nanjing China wangyuxin0212@126.com qghu@nju.edu.cn; School of Medical Imaging, Bengbu Medical College Bengbu 233030 Anhui China wangjianquan006@163.com

## Abstract

A multimodal drug delivery system targeting the tumor microenvironment is an inspiring method for treating cancer tissues, including oral squamous cell carcinomas (OSCC). Such approaches require an efficient and safe drug carrier. Bovine milk derived exosomes are ideal because the source is adequate and have advantages of both synthetic and cell-mediated nano carriers. In the present study, we developed a pH/light sensitive drug system based on milk-exosomes for OSCC therapy. It was called exosome–doxorubicin–anthracene endoperoxide derivative (Exo@Dox–EPT1, NPs). Milk-exosomes were conjugated to doxorubicin (Dox) by a pH-cleavable bond, which can rapture under an acidic microenvironment. Besides, endoperoxides and chlorin e6 (Ce6) were also loaded and the endoperoxides undergo thermal cycloreversion and release singlet oxygen to kill cancer cells. We have also investigated the body distribution, antitumor effects, and biocompatibility of the nanoparticles. The new milk-exosome-based drug delivery system showed controlled drug-release, biocompatibility and, proved to be effective in treating OSCC.

## Abstract 2

A multimodal drug delivery system targeting the tumor microenvironment is an inspiring method for treating cancer tissues, including oral squamous cell carcinomas (OSCC).

## Graphical abstract

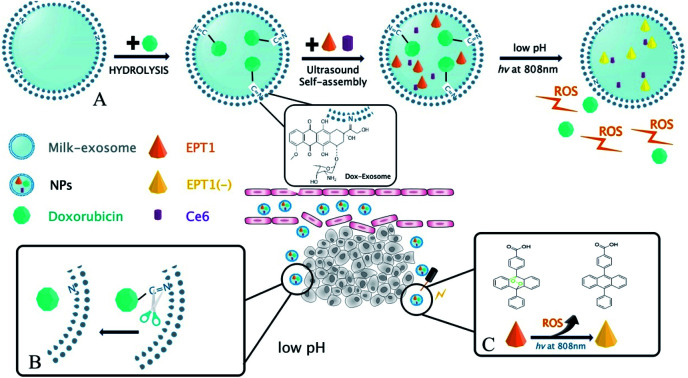

## 1. Introduction

Oral squamous cell carcinoma (OSCC) is the most common cancer in the head and neck region.^1^ Doxorubicin (Dox), paclitaxel and cisplatin are the first-line chemotherapy drugs for treating various cancers including OSCC.^2^ However, free agents usually require high dosage and result in unexpected systemic toxicity.^3^ Recently, nanomedicine advancement in cancer has provided great opportunities to solve these limitations.^4^ Nanoparticle-based drug delivery systems could accumulate in tumors through the enhanced permeability and retention (EPR) effect. Moreover, such drug delivery system encapsulated multifunctional drugs could be modified with cancer-associated biomarkers to realize active targets.^5,6^

Exosomes are lipid bounded natural vesicles with sizes between 40–100 nm. They are secreted by almost all kinds of cells through biological fluids, including blood plasma, urine, sweat and milk.^7,8^ In the intercellular cross-talking, exosomes have a vital role for transmitting signaling.^8,9^ Due to the characteristics that combine synthetic nano-carriers and cell-mediated vehicles,^10,11^ they are identified as an ideal natural drug delivery carrier. Currently, more and more types of exosomes or exosome-mimics have been exploited.^12–14^ For example, tumor cell-derived exosomes have been applied to treat malignant ascites and pleural effusion in a phase II study.^12^ More meaningfully, dendritic cells (DCs)-derived exosomes have entered in clinical trial to treat melanoma or small-cell lung carcinoma patients based on mechanism of antigen–antibody reaction.^15,16^ Therefore, exosomes have great potential to realize clinical translation. However, a number of disadvantages would impede the translation of autologous exosomes, such as limited yields, longer drug preparation time, cancer-stimulating risk and ethical problems.^11,17^

To overcome these deficiencies, bovine milk-derived exosomes were firstly isolated in 2010 (ref. 18) and gradually developed to potential drug delivery vehicle to its availability, cost and non-toxicity.^10^ The carrier has been proved to reduce systemic toxicity of free Dox^19^ and enhance drug accumulation in tumor tissues. It was reported that bovine milk-derived exosomes loaded with chemotherapeutic drugs showed better antitumor effects against lung and breast cancer.^10,20^ More importantly, oral chemotherapy might be realized in future because bovine milk-derived exosomes could pass through the gastrointestinal barrier.^21^ However due to its passive targeting, the exosomes loaded chemotherapeutic drugs would massively accumulate in various organs and cause possible damage to liver, kidney or heart.^16^ Therefore, it is necessary to improve the tumor-targeting of exosomes through the active targeting strategy. Alvarez *et al.* suggested that DCs-derived exosomes modified by neuron-specific RVG peptide and loaded with therapeutic siRNA were applied to treat Alzheimer's disease in a mouse model.^22^ Similarly, exosomes from immature DCs adapted by iRGD increased Dox delivery efficiency into breast cancer cells.^23^ In 2015, Gupta *et al.* firstly reported that milk-exosomes functionalized with folic acid improved efficacy and safety of drugs for cancer with high expression of folic acid receptors.^10^

Targeting acidic tumor microenvironment (TME) is a promising approach to engineer exosomes, except biomarker-targeting strategies. As we know, majority of solid tumors exhibit pH from 6.5 to 7.4, thus it give us possibility to fabricate PH-response nanoparticles.^24^ The imine bond is easily disintegrated in an acidic solution (pH = 6.8).^25^ Thus, if exosome membrane is conjugated chemotherapy drugs with hydrazone bond, the cleavage of the imine bond triggered by acidic TME would result in quick release of drugs in the tumor site. Next, hypoxia is another the distinct hallmarks of TME that induce irreversible tumor metastasis, as well as inflict hypoxia-associated resistance.^26^ Correcting the hypoxic TME with photodynamic therapy (PDT) is an effective strategies to treat cancer.^27^ The reactive oxygen (ROS) generated in PDT could destroy hypoxia in TME and kill cancer cells.^26^ Endoperoxide is one of the most reliable source of singlet oxygen^28^ and can generate singlet oxygen when temperature rise. Under near-infrared (NIR) light irradiation, photosensitizer-chlorin e6 (Ce6) produces plasmonic heat which results in singlet oxygen generation of endoperoxide. In general, targeting acid and hypoxic TME would achieve controlled release of chemotherapy drugs and photochemistry therapy.

**Fig. 1:**
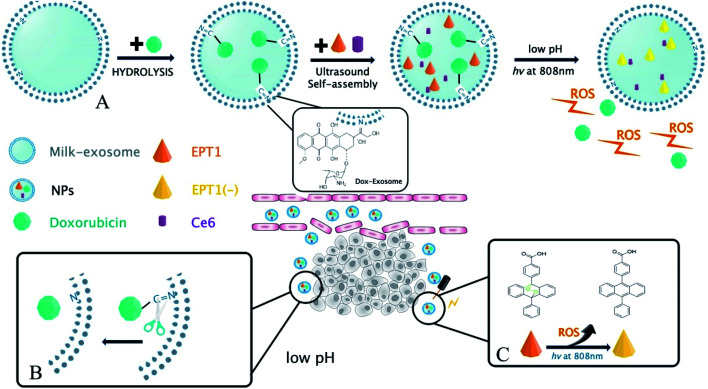
(A) Schematic illustration of the synthesis process for Exo@Dox–EPT1 (NPs). (B) Therapeutic mechanism of NPs under acidic tumor microenvironment. (C) PTT mechanism of NP under 808 nm near-IR light irradiation.

In the present study, a novel PH-response nanoparticles based on the bovine milk-derived exosomes as carrier were synthesized against oral squamous cancer cell (OSCC). First, Dox was modified on the exosomes membrane with imine bond, and then the tumor site-specific release of Dox was stimulated by PH in TME. Next, anthracene endoperoxide derivative (EPT1) and chlorin e6 (Ce6) were encapsulated in milk-exosome. When the nanoparticles were in tumor site, Ce6 produced plasmonic heat and accelerated singlet oxygen generation from EPT1 under NIR irradiation. Consequently, a reliable and tumor site-specific photochemistry therapy was realized in OSCC treatment. To our knowledge, our study might be the first report that the bovine milk-derived exosomes as carriers were applied in photochemistry synergistic therapy triggered by PH in TME and NIR irradiation. On the other hand, hematological assays and histopathology indicated that the nanoparticles had well biocompatibility *in vivo* without NIR stimulation. Therefore, we believed that the nanoparticles might be a promising prospect against OSCC. The main scheme of our study is depicted in Fig. 1.

## 2. Experimental section

### 2.1 Cell culture

Three human head and neck squamous cell carcinoma cell lines (HSC-3, SCC-9, CAL-27) and a human cardiac muscle cell line HCM were used in this study. The HSC-3 cell line was obtained from Fudan University, Shanghai, China while all other 3 cell lines were purchased from the American Type Culture Collection (ATCC, Manassas, VA). All cells were propagated in the conditioned (exosome free) Dulbecco's modified Eagle's medium (DMEM) (Gibco, Life Technologies, NY, USA) with fetal bovine serum (10%) and penicillin/streptomycin (100 units per ml). All reagents were purchased from Gibco, Life Technologies. Cells were maintained in an incubator at 37 °C in the presence of 5% CO_2_. Medium was renewed every 48 hours. Cells were used when grown to logarithmic phase.

### 2.2 Bovine milk exosomes isolation

Fresh unpasteurized milk (2 liters) was collected from the Weizhu Dairy located in Nanjing, China and stored at 4 °C for 2 hours before the isolation of exosome.

Exosomes were isolated from milk using the differential centrifugation as described previously.^10,29^ Briefly, milk was centrifuged at 3000 × *g* for 15 min at 4 °C to remove fat globules, cellular debris and somatic cells. The whey was collected by passing through a cheese cloth and transferred into polycarbonate tubes. In order to remove large particles, it was centrifuged for 60 min at 100 000*g* in Type 45Ti fixed angle rotor using Optima LE-80K Ultracentrifuge (Beckman Coulter, Brea, California). Supernatant was finally centrifuged at 135 000 × *g* for 90 min at 4 °C. Then the final supernatant was discarded and the exosome pellet was washed twice using the phosphate-buffered saline (PBS). The exosome pellet was re-suspended in PBS, followed by filtration through a 0.22 μm Steritop filter (Millipore, Darmstadt, Germany). The total protein concentration was determined using the bicinchoninic acid (BCA) kit (Beyotime Biotechnology, China). The concentration of the milk derived exosomes was adjusted to 6 mg ml^−1^ and stored at −80 °C until further use. The isolated exosomes were characterized as recommended by the International Society of Extra Cellular Vesicles (ISEV).^30^

### 2.3 Synthesis of Exo@Dox–EPT1 (NPs)

#### 2.3.1 Synthesis of EPT1

The preparation of 9-carboxyphenyl-10-phenylanthracene (carboxy-EPO, EPT1) was performed following the method described previously.^28^ The structure of EPT1 was further confirmed through nuclear magnetic resonance (HNMR, CNMR) spectroscopy and UV-vis spectroscopy.

#### 2.3.2 Synthesis of pH sensitive Exo@DOX

Doxorubicin could be conjugated to milk-exosomes through C=N bond, as previously reported by Nie Shuming.^31^ In brief, milk-exosome was lyophilized at −4 °C for further use. Doxorubicin (10 mg/0.017 mmol) dissolved in 10 ml of ethanol was added to triethylamine (10 μl/2 mg) and stirred at room temperature for 24 hours. The solution obtained was filtered to further react with milk-exosome at 60 °C for 24 h. Then it was purified for 24 hours using dialyses against cellulose membranes (*M*_w_ = 3.5 kDa) in PBS at 4 °C to remove unreacted compound.

#### 2.3.3 Synthesis of Exo@DOX–EPT1 (NPs)

The prepared solution of Exo@DOX–NPs was mixed with 5 mg of EPT1 and Ce6 (1 mg) and oscillated ultrasonically oscillation for 5 hours. The unloaded EPT1 and Ce6 were removed by passing through the disposable PD-10 desalting columns. The resulting solution was directly used without any further treatment. Synthesis process and therapy mechanism was illustrated in Fig. 1.

### 2.4 Transmission electron microscopy (TEM)

**Fig. 2:**
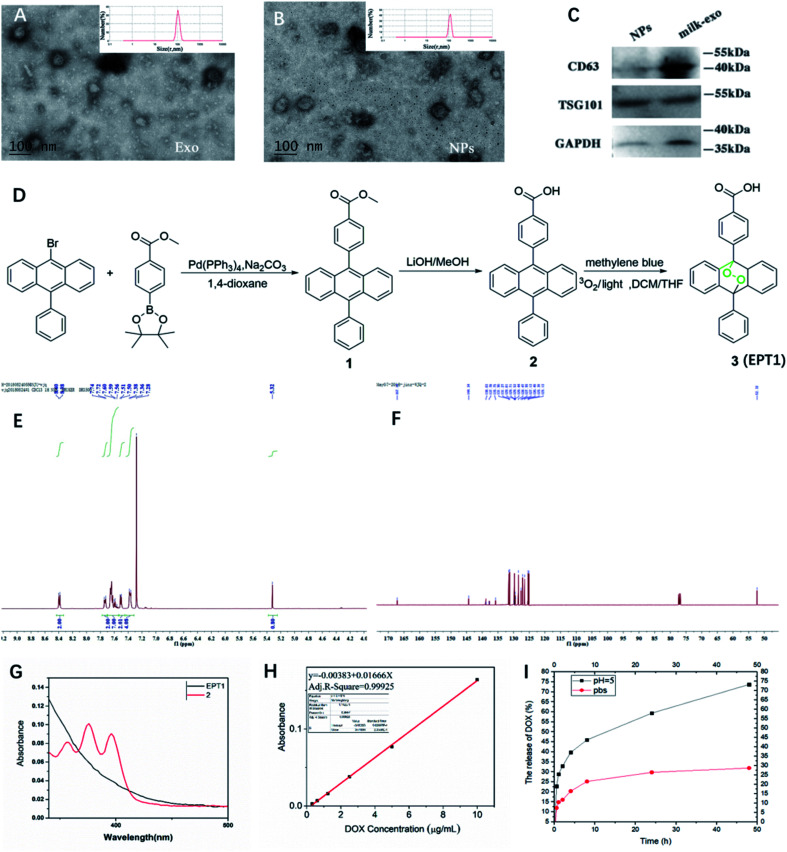
Characterization of the milk-exosome and Exo@Dox–EPT1 (NPs). TEM images and size distribution of milk-exosomes (A) and NPs (B); TEM scale bar, 100 nm. (C) Western blotting analysis of CD63 and Tsg101 in milk-exosome and NPs. (D) The synthetic route of EPT1. (E) ^1^H-NMR spectrum of EPT1. (F) CNMR spectrum of EPT1. (G) The UV-vis absorption spectra of EPT1 and compound 2. (F) ^1^H-NMR spectrum of EPT1. (H) Standard absorption curve of Dox. (I) *In vitro* release of Dox from the NPs at 37 °C in PBS (pH = 7.4) and acetate buffer (pH = 5.0).

Milk-derived exosomes (6 mg ml^−1^) were diluted to 1000 folds using deionized water and added onto the clean silica wafer and lyophilized. The extracted milk-exosome and NPs were characterized using a TEM (JEM-2010, JEOL, Japan) at an acceleration voltage of 80 kV (Fig. 2A and B). 10 μl sample was pipetted onto the formvar/carbon-coated nickel grid, and stained by phosphotungstic acid (PTA, 1%) solution.

### 2.5 Dynamic light scattering (DLS)

Hydrodynamic particle size analysis was performed using dynamic light scattering by ZetaSizer Nano-ZS90 (Malvern Instrument, Worcestershire, United Kingdom). Purified milk-exosomes and synthetic NPs were resuspended in 1 ml PBS and analyzed for particle size distribution.

### 2.6 Western blot analysis

Milk-derived exosomes for western blotting were suspended in 100 μl RIPA buffer (Solarbio, Shanghai, China). The exosomal surface proteins were analyzed by western blotting as described previously.^10^ Blots were probed for CD9, CD63, Tsg101 (Abcam, Cambridge, UK) using secondary antibody anti-GM130 (Abcam, Cambridge, UK). All antibodies were used following the manufacturer's instructions.

### 2.7 Drug loading and encapsulation

The encapsulation efficiency and drug loading content of Dox were calculated using the following equation;Drug loading content of Dox = total amount of Dox − weight of free Dox/weight of Dox–NPs

### 2.8 *In vitro* uptake of NPs

Because Dox is intrinsically fluorescent, uptake of free Dox or NPs can be observed at 594 nm using confocal microscopy after excitation at 480 nm. Briefly, HSC-3, SCC-9, CAL-27 and HCM cells were seeded into a 6-well plate at a density of 1 × 10^5^ cells per well. All cells treated with free Dox or NPs (5 μg per well, Dox). Cells were labeled with fluorescent probe Dil (Beyotime Biotechnology, China) after 8 hours of treatment, and *in vitro* uptake was calculated detected by a confocal laser scanning microscopy (Carl Zeiss Microscope Systems, Jena, Germany).

### 2.9 *In vitro* release ability of Dox

To measure the release of Dox from NPs in response to pH stimuli, NPs were dispersed in the PBS (pH = 7.4) and acetate buffer (pH = 5.0) respectively. NPs solution (1 ml) in a dialysis bag (*M*_w_ = 1 kDa) was added to 10 ml of PBS (pH 7.4) in a container and vibrated using a thermostatic oscillator at 37 °C for 48 h. An aliquot of dialysate (1 ml) was taken at 1 h, 2 h, 4 h, 8 h, 12 h, 24 h, and 48 h respectively. Simultaneously, PBS was added into the dialysis bag having 1 ml of remaining dialysate. The amount of released Dox was measured by the standard curve of Dox. The amount of released Dox under acidic conditions was similar to the above-mentioned operation.

### 2.10 *In vitro* release ability of ROS

The HSC-3, SCC-9, CAL-27 and HCM cells (1 × 10^5^ cells per well) were treated with PBS, H_2_O_2_ and NPs containing 3 μg of equivalent Dox per well. Two plates of each cells were treated with NPs, amongst only one plate received additional light excitation at 808 nm for 3 minutes. After 6 hours, treated cells were washed using Hank's balanced salt solution (HBSS), and incubated for 30 min at 37 °C with 10 μM 2′,7′-dichlorofluorescein diacetate (DCFDA) dye (Molecular Probes, NY). Cells treated with PBS and incubated with DCFDA were used as controls. Cells were collected and ROS levels were determined by measuring the fluorescence using a confocal laser scanning microscopy (Carl Zeiss Microscope Systems, Jena, Germany) at 485 nm excitation and 535 nm emission wavelength. Cells treated with H_2_O_2_ (1 : 1000) were set as positive control.

### 2.11 *In vitro* cytotoxicity assay

The cytotoxicity of NPs on HSC-3, SCC-9, CAL-27 and HCM cells was tested using the Cell Counting Kit-8 (CCK-8) assay (MedChem Express, USA). Briefly, cells were seeded into a 96-well plate overnight until the confluence reached 80–85%. Then, the medium was replaced by 100 μl of milk-exosomes, free Dox or NPs (with equivalent Dox 0.5 mg ml^−1^) for 6 hours. One plate of cells was treated with NPs only while the other plate of cells was irritated at 808 nm for 3 min following NPs administration. After 24 hours, an aliquot of 10 μl of CCK-8 solution was added in each hole and incubated for another 2 hours in 5% CO_2_ at 37 °C. Absorbance at 450 nm was measured and cell viability was calculated based on the absorbance data.^32^

### 2.12 *In vivo* anti-tumor activity

Nude mice bearing HSC-3, SCC-9 and CAL-27 cancer cells respectively were administered several types of drugs intravenously including milk-exosome, free Dox or NPs when tumor volume reached 100 mm^3^. According to the drug administration, mice were divided into 5 groups (*n* = 5 for each group): a single dose of 100 μl milk-exosomes (60 mg kg^−1^ exosome protein), laser (after milk-exosomes injection), free Dox (0.25 mg kg^−1^, amount of Dox), NP_nl_ (0.25 mg kg^−1^, amount of Dox, no laser), and NP_808_ (0.25 mg kg^−1^, amount of Dox, laser). Drugs were administered every 3 days for 10 times and tumor volumes were recorded simultaneously. The 808 nm laser (1 W cm^−2^) was used in additional radiation for 3 min in laser and NP_808_ groups. The longest diameter (*L*) and shortest diameter (*S*) were measured every two days by the same observer. The tumor volume was calculated using the following formula: *V* = (*L* × *S*^2^)/2. All animal procedures were performed in accordance with the Guidelines for Care and Use of Laboratory Animals of Nanjing University and approved by the Animal Ethics Committee of Nanjing.

### 2.13 *In vivo* distribution

Athymic nude mice (*n* = 4 per group) bearing HSC-3 tumor were employed. Indocyanine green (ICG) encapsulated NPs were prepared and dispersed in 100 μl PBS. ICG-NPs were administrated into HSC-3 tumor bearing mice *via* an intravenous injection. *In vivo* fluorescence imaging was obtained at various time intervals (5 minutes, 1 hour, 2 hours, 6 hours, 12 hours, 24 hours and 48 hours). Then mice were executed to harvest major organs and tumor tissue for fluoresce imaging. All fluorescence images were obtained by *in vivo* fluorescence imaging system (Cri Inc., MA, USA).

### 2.14 Statistic analysis

Triplicate data were analyzed in GraphPad Prism 6 and shown as mean ± standard deviation (SD). Outcome variables were compared among treatment groups using the unpaired *t*-test and one-way analysis of variance (ANOVA). *P*-Values less than 0.05 were considered to be statistically significant.

## 3. Result and discussion

### 3.1 Characterization of milk-exosome and Exo@DOX–EPT1 NPs

The present study isolated and analyzed the milk-exosome and Exo@DOX–EPT1 NPs. Our data showed a typical exosomal characterization of milk-exosomes and NPs (Fig. 2). The diameter and size measurements of isolated vesicles and NPs using DLS indicated that the isolated milk-exosome and NPs after synthesis have an average diameter of 105.7 nm, and 122.4 nm respectively (Fig. 2A and B). The size and structural morphology of milk-exosome and NPs were confirmed by TEM and showing a typical bilayer membrane (Fig. 2A and B). In addition, the western blotting analysis confirmed the existence of typical membrane proteins of exosome, including CD63 and Tsg101 (Fig. 2C).

EPT1 is a reliable source of singlet oxygen that can release ROS under remote-controlled heating conditions independent from tissues' oxygen concentration. The anthracene endoperoxide can be easily monitored by UV-vis spectroscopy, as the endoperoxide does not have any absorption bands at wavelengths longer than 350 nm. In contrast, the corresponding anthracene has several diagnostic bands in the range of 350 nm to 425 nm.^28^ The synthetic route of EPT1 was illustrated in Fig. 2D and the structure of loaded EPT1 was confirmed through HNMR, CNMR and UV-vis spectroscopy analysis (Fig. 2E–G), and consistent with the literature report.^28^ The EPT1 drug loading content was 5.8% in this study.

### 3.2 PH-response Dox release

Dox concentration was calculated based on standard absorption curve as shown in Fig. 2H. The initial drug loading content of Dox was 13.4%. In order to verify Dox loading and pH-controlled release ability of NPs, they were dispersed in the PBS (pH = 7.4) and acetate buffer (ABS; pH = 5). Dox released from NPs in two different pH buffers was compared at variable time points (Fig. 2I). The results indicated that almost 25% Dox was released from NPs after 9 h of incubation and maintained a stable suspension in PBS. When pH was decreased to 5 (ABS), Dox release rate after 9 h was increased to 42.5% and almost 73% during 50 hours. These finding suggested that Dox can have sustained release from NPs under acidic conditions.

### 3.3 Release of ROS under light excitation

**Fig. 3:**
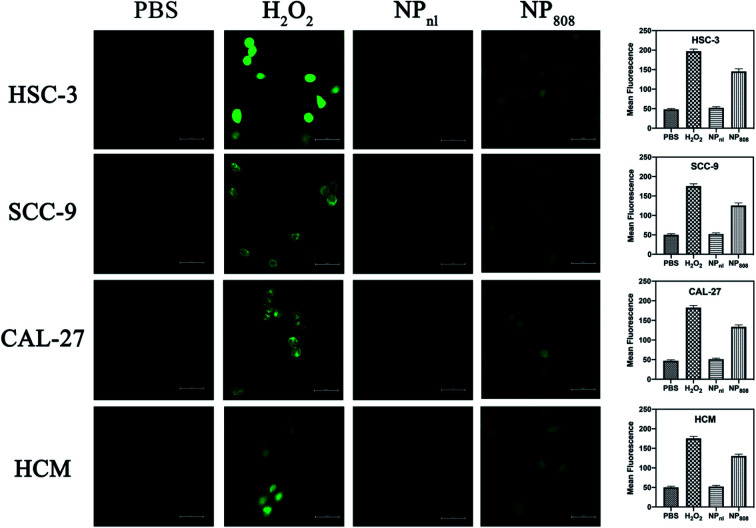
ROS generation detection of HSC-3, SCC-9, CAL-27 and HCM cells after treatment with various formulations. Cells were incubated with PBS, H_2_O_2_, and NPs (5 μg Dox per well). Cells treated with NPs were irradiated with 808 nm laser (2.0 W cm^2^, 3 min). Treated cells were then incubated with DCFDA dye (10 μM) for another 30 min at 37 °C for ROS detection.

As the ROS generation diminished corresponding to decreased cellular oxygen concentration, many PDT methods based on organic photosensitizer are self-limiting (*e.g.* porphyrin,^33^ phthalocyanine,^34^ chlorine^35^). In the present study, we built up a novel PDT system based on milk-exosome loaded anthracene endoperoxide derivative and Ce6. HSC-3, SCC-9, CAL-27 and HCM cells were treated with PBS, H_2_O_2_ and NPs with or without irritation at 808 nm. The ROS production of different samples was analyzed using DCFD kit. NPs released no apparent ROS without light irritation, but produced ROS effectively after irritation with an 808 nm NIR laser (1 W cm^−2^) (Fig. 3). These results proved the PDT ability of the fabricated NPs.

### 3.4 Cell uptake

**Fig. 4:**
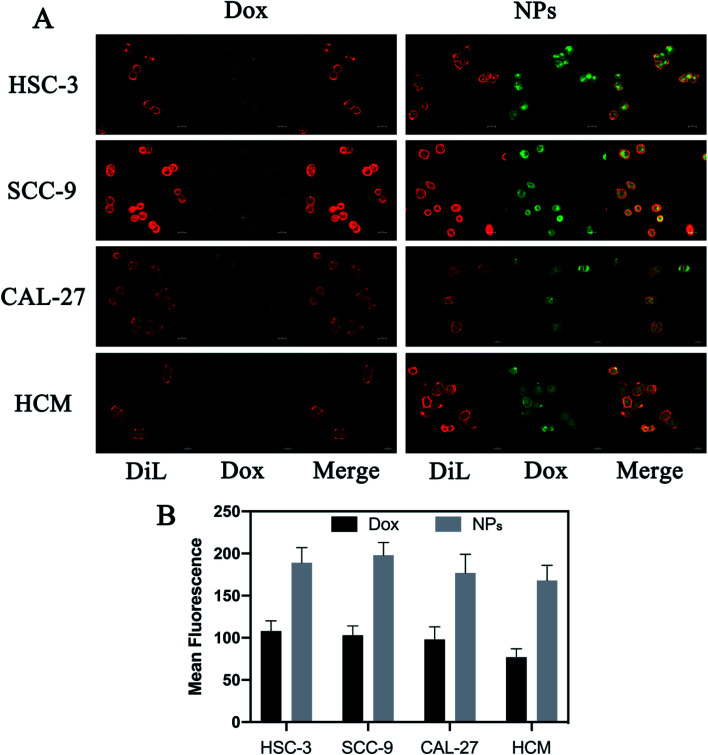
Fluoresce microscopy comparing the uptake of NPs and free Dox by HSC-3, SCC-9, CAL-27 and HCM cells. All cells were treated with free Dox or NPs (5 μg per well, Dox). 8 hours after treatment, cells were labeled by Dil probe and the uptake of free Dox or NPs were observed at 594 nm following excitation at 480 nm using confocal microscopy which showed more accumulation of NPs in the cytoplasm.

Drug delivery system based on exosomes has been demonstrated to deliver various therapeutic cargos that can easily be internalized by recipient cells.^36^ This study examined the uptake of NPs compared to free Dox using the confocal microscopy. Briefly, HSC-3, SCC-9, CAL-27 and HCM cells were stained with dye Cy5 and treated with free Dox or NPs for 8 hours (with equivalent Dox of 5 μg per well). The cell internalization was observed by detecting emission of Dox using the fluoresce microscopy. NPs-treated cells displayed more intense green fluorescence in the cytoplasm compared to free Dox-treated cells (Fig. 4). It indicated that more NPs were taken up *via* milk-exosomes.

### 3.5 Anti-tumor effects *in vitro*

**Fig. 5:**
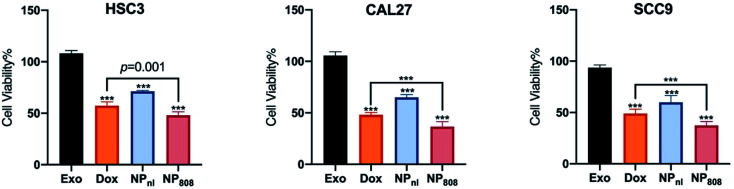
Cytotoxicity of HSC-3, SCC-9 and CAL-27 cancer cells at 6 h post incubation with different formulations. All cells were incubated with free Dox or NPs (equivalent Dox 0.5 mg ml^−1^). ***P < 0.0001.

The anti-tumor activity of NPs against HSC-3, SCC-9 and CAL-27 cancer cells was investigated using CCK-8 assay. All cancer cells were co-cultured with different samples for 6 hours, including Exo group (milk-exosomes treated only), free Dox group, NP_nl_ (no laser group, NPs with equivalent Dox 0.5 mg ml^−1^) and NP_808_ groups (laser irradiation, NPs with equivalent Dox 0.5 mg ml^−1^). No obvious cell death was observed in Exo groups (cell viability, HSC-3: 108.2%, CAL-27: 105.5%, SCC-9: 93.7%). After treatment of free Dox, 57.28% HSC-3 cells, 48.9% SCC-9 cells and 48.1% CAL-27 cells survived. In NP_nl_ group, 71.32% HSC-3 cells, 59.9% SCC-9 and 65% CAL-27 cells survived. Following treatment of NPs with 808 nm laser irradiation, cell viabilities of HSC-3 cell, SCC-9 cells and CAL-27 cells reduced to 48.02%, 37.43% and 36.57% respectively. Obviously, the NPs caused significant more cytotoxicity in all 3 cancer cells under 808 nm laser irradiation (1 W cm^−2^) than free Dox group (*P* < 0.05) (Fig. 5).

### 3.6 Antitumor effect *in vivo*

**Fig. 6:**
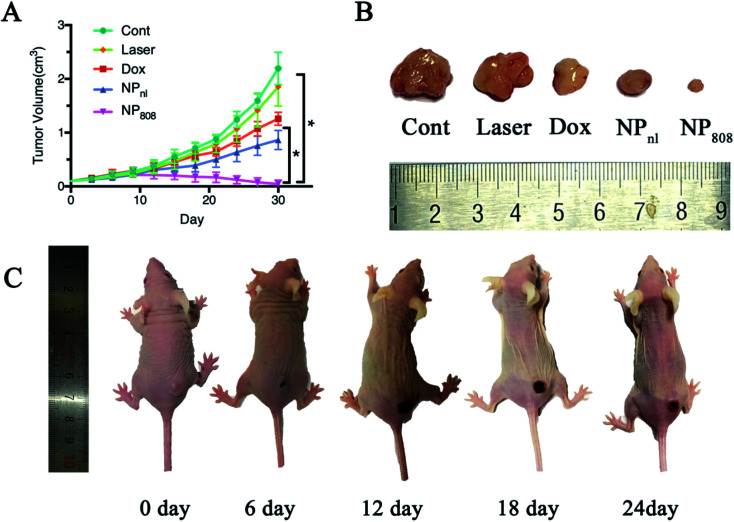
Antitumor effects *in vivo*. (A) Tumor volumes of mice receiving different treatments; (B) images of tumors harvested from each group; (C) *in vivo* antitumor effects following the injection of NP and 808 nm irradiation. *P < 0.05.

To further evaluate the therapeutic effects of NPs *in vivo* against OSCC, mice bearing HSC-3 xenograft tumors were randomly divided into five groups (five mice for each group) when tumor sizes reached approximately 100 mm^3^. The anti-tumor effect of different samples clearly showed that the mice treated with milk-exosomes (control) had the fastest tumor growth and reached the largest volume about 2.19 (SD = 0.30) cm^3^ at the end (Fig. 6). Mice treated with laser revealed no obvious changes in the tumor growth compared in the control group mice (1.85 cm^3^, SD = 0.36 of tumor volume at the end, *P* > 0.05). In case of mice treated with free Dox and NP_nl_, tumor growth was significantly slower and reached 1.26 cm^3^ (SD = 0.12) and 0.86 cm^3^ (SD = 0.18) respectively. Moreover, in group NP_808_, the tumor growth was restrained effectively and almost disappeared after treatment (average tumor volume 0.05 cm^3^, SD = 0.07). Therefore, the NPs produce synergistic effects of photochemistry which can be triggered by acid TME and NIR and is a novel promising therapeutic approach against OSCC.

### 3.7 Biodistribution *in vivo*

**Fig. 7:**
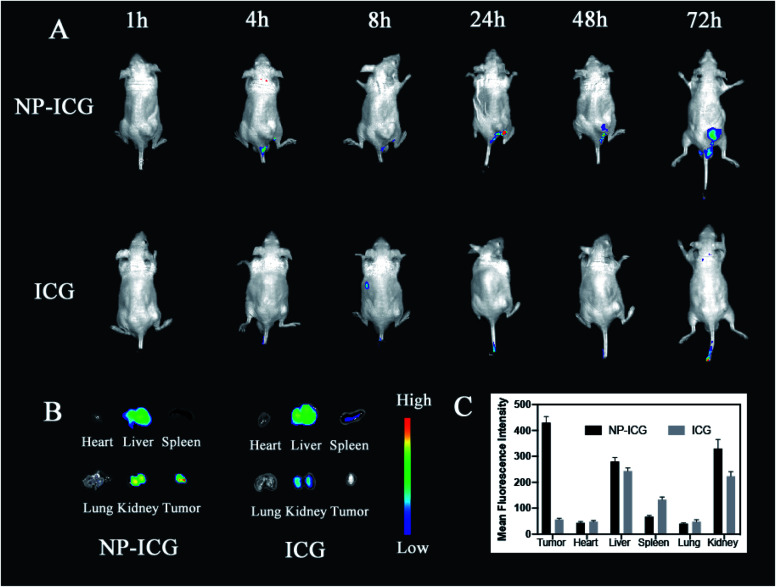
Fluorescent imaging of tissues *in vivo*. NPs were labeled with ICG for *in vivo* imaging. Mouse was injected with free ICG or NP@ICG and dynamic *in vivo* fluorescent imaging was taken for 72 h (A). Organ was harvested at 72 h after intravenous injection of NP@ICG or free ICG (B). It showed more accumulation of NPs in tumor tissues for a longer period of time compared to free ICG. (C) Biodistribution of free ICG and NP-ICG in mice determined from the ICG FL images. The data are shown as mean ± SD (*n* = 4).

In order to examine the *in vivo* distribution of the NPs, we created ICG-labeled NPs for *in vivo* fluorescence imaging experiments. An aliquot of 100 μl of free ICG (control) and NPs was injected intravenously into HSC-3 tumor-bearing nude mice with an equivalent dose of ICG (0.3 mg ml^−1^). The fluorescent imaging was performed at 1, 4, 8, 24, 48, and 72 hours after injecting the medicaments (Fig. 7). At 24 hours after injection, fluorescent signals of tumor tissues in NP@ICG group were stronger than that of free ICG group. Moreover, NP@ICG still had remarkable accumulation in tumor tissues at 72 hours while fluorescent signals of free ICG group were invisible in tumor at the same time. After 72 hours, the mice were euthanised and their organs and tumor tissues were harvested for fluorescent imaging analysis. Strong signals were observed in the liver and kidney tissues in both groups. Furthermore, the NP@ICG group had much more fluorescent intensity in tumor while that of free ICG group was invisible. In summary, NPs enhanced the drug accumulation in tumor tissues and had longer retention time *in vivo*.

### 3.8 Biocompatibility

**Fig. 8:**
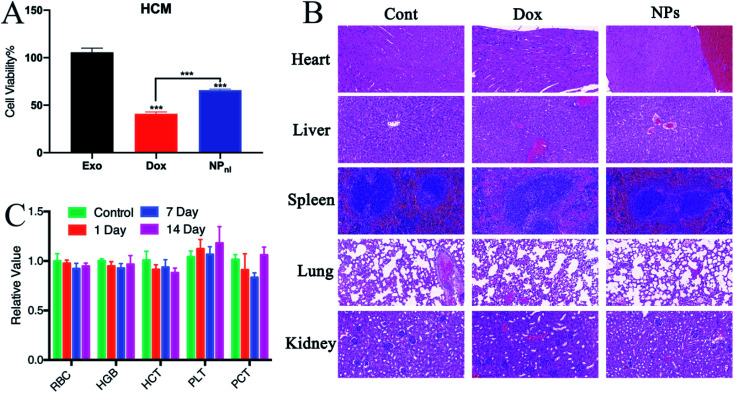
Biocompatibility assessment using various compounds. (A) Cytotoxicity analysis revealed more numbers of HCM cells were survived in NPs group. (B) Histopathology (H&E staining) showed obvious myocardial damage following the Dox injection, including myocardial hypertrophy and tissue degeneration. (C) Hematology assay suggested no abnormality after NPs injection at 1, 7 and 14 days.

Non-specific toxicity is one of the major limitation of traditional chemotherapeutic agents, including Dox.^4^ The cardiotoxicity of Dox is a major complication for clinical treatment. Exosome-based drug delivery system was proven to reduce normal tissues toxicity from Dox.^19^ In cytotoxicity evaluation, this study indicated that more numbers of HCM cells were survived after treated with NPs alone than free Dox (Fig. 8A). To test the biocompatibility of NPs *in vivo*, mice received intravenous injection of Dox or NPs every 3 days at equivalent quantities. Mice treated with PBS were set as control group. The hematology assay for mice treated with NPs or PBS were administrated every 7 days. The histopathology of major organs (heart, liver, spleen, lung and kidney) were further performed. In mice treated with Dox, the pathological examination clearly showed myocardial damage including cardiomyocyte hypertrophy and myocardial tissue degeneration (Fig. 8B). However, no apparent hematological abnormality (Fig. 8C) and obvious pathological change were observed in mice treated with NPs. The results suggested that NPs had a good biocompatibility *in vivo*.

## 4. Conclusions

The study might be the first study that a new sensitive drug delivery system based on milk-exosomes for photochemistry therapy against oral squamous cell carcinoma. Dox was modified on the exosomes membrane with imine bond, and then the tumor site-specific release of Dox was stimulated by acid environment. Next, EPT1 and Ce6 were encapsulated in milk-exosome. When the NPs were accumulated in tumor site, Ce6 produced plasmonic heat and accelerated ROS generation from EPT1 under NIR irradiation. Therefore, the photochemistry therapy against OSCC was realized based on the pH/light sensitive Exo@Dox–EPT1. *In vitro* and *in vivo* testing supported well the biocompatibility of this Exo@Dox–EPT1. Consequently, the Exo@Dox–EPT1 might be a promising prospect to treat OSCC.

## Conflicts of interest

The authors declare no competing financial interest.

## Supplementary Material
